# Draft Genome Sequence of Clostridium perfringens Strain TAMU, Which Causes Necrotic Enteritis in Broiler Chickens

**DOI:** 10.1128/MRA.01357-19

**Published:** 2020-01-23

**Authors:** Catherine Ausland, Adil Sabr Al-Ogaili, Juan D. Latorre, Guillermo Tellez-Isaias, Billy M. Hargis, Young Min Kwon, Margarita A. Arreguin-Nava, Pallavi Singh

**Affiliations:** a Department of Biological Sciences, Northern Illinois University, DeKalb, Illinois, USA; b Department of Poultry Science, University of Arkansas, Fayetteville, Arkansas, USA; c Department of Medical Laboratory Techniques, Kut Tech Institute, Middle Technical University, Baghdad, Iraq; d Eco-Bio, LLC, Fayetteville, Arkansas, USA; University of Maryland School of Medicine

## Abstract

Clostridium perfringens causes severe gastrointestinal diseases, which include necrotic enteritis (NE) in chickens, a deadly disease worldwide. We report here the draft genome sequence of Clostridium perfringens strain TAMU, which was used in developing an NE chicken challenge model. This C. perfringens TAMU genome sequence will aid in advancing potential intervention strategies to reduce NE pathogenesis.

## ANNOUNCEMENT

Clostridium perfringens, a Gram-positive spore-forming bacterium, is the causative agent of an array of diseases in humans and agricultural animals due to diverse toxins on its conjugative plasmids (1–4). One of these diseases is necrotic enteritis (NE), which causes intestinal inflammation and necrotic regions in broiler chickens, leading to high morbidity and mortality. C. perfringens has also been isolated from commercial turkeys with cellulitis (5). NE is increasingly a significant burden on the poultry industry, particularly with the banning of prophylactic antibiotic use (6). Here, we present the draft genome sequence of C. perfringens strain TAMU, which was isolated in 2004 using a brucella blood agar plate from the gut of a broiler chicken from Texas presenting NE (7, 8). Since then, our group has successfully used the C. perfringens strain to reproduce important aspects of NE pathogenesis using an *in vitro* digestive model (9) and *in vivo* chicken challenge models (8, 10, 11).

C. perfringens TAMU was cultured overnight in tryptic soy broth with thioglycolate, and DNA extraction was performed with a DNeasy UltraClean microbial kit (Qiagen LLC, Germantown, MD). Genomic DNA was prepared for shotgun metagenome sequencing using a Nextera XT DNA library preparation kit (Illumina, Inc., San Diego, CA) according to the manufacturer’s instructions. Sequencing was performed at the University of Illinois at Chicago Sequencing Core (UICSQC) using a NextSeq 500 instrument (Illumina, Inc.) with 150-bp paired-end sequencing. In total, approximately 6.1 million reads were generated. Trimming was performed in the software package CLC Genomics Workbench v11.0.1 (Qiagen). Trimming was performed using default parameters with a threshold of Q20. Sequences demultiplexed in the BaseSpace cloud computing environment provided by the UICSQC resulted in a 3,672,352-bp draft genome assembly using SPAdes v3.11.1 (12) with 318 contigs more than 200 bp in length, an average coverage of 205×, an *N*_50_ value of 46,751 bp, and a G+C content of 28.11%. Genome assembly quality was determined by the QUAST quality assessment tool (13).

The draft genome was annotated with the Prokaryotic Genome Annotation Pipeline from NCBI (14). Annotated features include 3,397 genes with 3,309 coding sequences (CDS), 12 rRNAs (including 5S, 16S, and 23S rRNAs), 75 tRNAs, and 4 noncoding RNAs (ncRNAs). Functional annotation with the Virulence Factors Database (VFDB) (15) predicted 25 open reading frames (ORFs) associated with virulence, including several encoding enterotoxins (*n* = 3), hemolysins (*n* = 4), and adherence factors (*n* = 2), as well as alpha-toxin (*n* = 1) and β2-toxin (*n* = 1). These data have been made available on figshare (https://doi.org/10.6084/m9.figshare.11337245.v3). The Resistance Gene Identifier in the Comprehensive Antibiotic Resistance Database (16) identified 3 genes conferring resistance to peptide (*n* = 1) and tetracycline (*n* = 2) antibiotics based on protein homology models of these genes sharing 97% or greater similarity to query sequences (https://doi.org/10.6084/m9.figshare.11337311.v1). Default parameters were used for all software unless otherwise specified. In conclusion, this C. perfringens TAMU draft genome sequence will faciliate functional genomic analysis of virulence factors associated with NE pathogenesis.

### Data availability.

This whole-genome shotgun project has been deposited at DDBJ/EMBL/GenBank under the accession number VOVJ00000000. The version described in this paper is version VOVJ01000000. The project data have been submitted under BioProject accession number PRJNA558493 and raw sequences under SRA accession number SRP218148.
